# The Effect of Physical Activity Levels on Cognitive Performance: Research in Portuguese Adolescents

**DOI:** 10.3390/sports12060146

**Published:** 2024-05-27

**Authors:** Ana Rodrigues, Hélio Antunes, Bebiana Sabino, Duarte Sousa, Ana Luísa Correia, Ricardo Alves, Hélder Lopes

**Affiliations:** 1 Department of Physical Education and Sport, Faculty of Social Sciences, University of Madeira, 9020-105 Funchal, Portugal; anajar@staff.uma.pt (A.R.); h.antunes@staff.uma.pt (H.A.); duartes@staff.uma.pt (D.S.); anacorreia@staff.uma.pt (A.L.C.); ricardo.alves@staff.uma.pt (R.A.); 2 Research Center in Sports Sciences, Health Sciences, and Human Development (CIDESD), 5001-801 Vila Real, Portugal; 3 Higher School of Education, Polytechnic Institute of Beja, 7800-295 Beja, Portugal; bebiana.sabino@ipbeja.pt; 4 SPRINT Sport Physical Activity and Health Research & Innovation Center, 4900-367 Viana do Castelo, Portugal

**Keywords:** cognitive performance, physical activity, accelerometry, adolescents

## Abstract

The literature unequivocally acknowledges the numerous health benefits that physical activity (PA) provides. However, in other variables, such as cognitive performance (CP), the PA characteristics required to elicit favorable benefits remain controversial, particularly among adolescents. The aim was to investigate the evolution of CP in adolescents over the school year, as well as the role of regular PA levels. The study included 366 adolescents (boys *n* = 154), between 12 and 20 years old (15.46 ± 1.63), from middle school *(n* = 123) and high school (*n* = 243). CP was assessed through a face-to-face interview employing the Cognitive Telephone Screening Instrument. The variation in CP (∆CP) was determined by the difference between the value of the final assessment (end of the school year) and the initial assessment (start of the school year). PA was assessed using accelerometry (ActiGraph GT3X+). The CP score improved from the initial to the final assessment (37.80 ± 9.26 vs. 40.45 ± 10.05) (t = −6.135; *p* < 0.001; Glass’s Delta = 0.37. Multiple linear regression revealed that age (ß = −0.332; t = −4.255; *p* < 0.001) and high-intensity PA (ß = 0.283; t = 3.627; *p* < 0.001) accounted for 17.2% of the variation in ∆CP. CP improved significantly over the school year, emphasizing the significance of age and vigorous PA in ∆CP in adolescents.

## 1. Introduction

Physical activity (PA) is essential for promoting public health and individual well-being. However, physical inactivity continues to be the foremost global risk factor for mortality and morbidity [1]. Regular PA has numerous benefits for both our physical and emotional well-being. These include improved psychological wellness [2], weight management, a decreased risk of disease, stronger bones and muscles, and an increased capacity to do daily tasks [3]. Similarly, regular PA in adolescence yields several health benefits and can contribute significantly to long-term well-being. Studies have found that childhood obesity is a powerful predictor of adult obesity and the development of chronic diseases, such as diabetes [4]. Furthermore, regular PA in adolescence emerges as a pivotal approach in attenuating the risk factors for a variety of health issues, including heart disease, obesity [5], type 2 diabetes, and mental health [6].

The World Health Organization recommends that children and adolescents participate in at least 60 min of moderate to vigorous intensity PA per day to improve their health [7]. Despite the evidence of health benefits associated with adhering to these benefits, many countries, including Portugal [8], employ strategies that prove ineffective in addressing physical inactivity [9]. Overall, 81% of adolescents do not comply with these recommendations [10]. According to studies conducted on Portuguese adolescents, only 15 to 30% of the teens follow the PA recommendations [11,12].

Schools wield a distinctive capacity to encourage healthy lifestyles owing to their inherent mission and the substantial amount of time children and adolescents spend in this setting [13]. However, this is not being valued. Contemporary professional demands have led to a heightened emphasis on particular classes within the curriculum at the expense of recognizing possibilities for active student behavior, such as physical education [14]. This singular focus on excellent academic success overlooks compelling scientific findings regarding the relationship between physical education/school-based PA and academic performance [14,15,16]. Moreover, research has shown that regular PA in children and adolescents is associated with improved cognitive skills, including attention, memory, and executive functions, such as problem-solving and decision-making [17]. These domains have been positively related to academic performance [18].

A link between PA and CP seems evident in the literature; however, the type/domain, duration, intensity, and frequency of PA should be taken into account when evaluating its effects on CP [19]. Despite extensive research, determining the optimal dose of PA remains inconclusive for adolescents. Considering PA type, a review of 58 publications indicated that sports engagement improves CP and well-being [17]. Young people involved in sports demonstrate better performance in learning and memory tasks compared to those who do not play sports [20,21]. Simultaneously, it has been shown that competitive young athletes exhibit enhanced mental imagery skills compared to non-athletes [22]. Additionally, literature reports that active transportation to school for more than 30 min also improves cognitive function, especially in inductive reasoning and working memory dimensions [23]. Another systematic review indicated that PA, particularly aerobic activity, promotes cardiovascular performance, which is associated with enhanced cognitive functioning [24,25]. However, detailed insights into the frequency and intensity of PA predominantly focus on the elderly population. Higher intensity of PA indicates an increase in the brain’s functional connectivity, particularly the intra-subcortical and frontal-subcortical networks, which are linked to cognitive processes like memory [26]. Changes in brain function and structure [27], such as increased gray matter volume in the hippocampus and frontal cortex [28], intensified blood flow, neural transmission, and neurotrophic factors [27] also appear to be associated with vigorous PA.

Another small number of studies carried out with children show that low to high-intensity physical activity provides immediate gains in executive function [29,30]. Nevertheless, few studies have delved into determining the optimal level of PA that exerts the highest impact on CP among adolescents.

Therefore, the primary goal of this study is twofold: (i) assess the progression of CP among adolescents over the course of a school year, and (ii) determine if the intensity of habitual PA affects the fluctuations in CP throughout the school year.

## 2. Materials and Methods

This is a longitudinal study, with participants being assessed on cognitive function at the beginning (September 2022) and at the end of the school year (May 2023). Concurrently, they were assessed for physical activity using accelerometry throughout a standard school week, encompassing five weekdays and two weekends.

### 2.1. Sample

The sample in this study is part of a larger research project “Physical Education in Schools in the Autonomous Region of Madeira” (EFERAM-CIT).

A total of 366 subjects participated in the study (male = 154 and female = 212), aged between 12 and 20 years (15.46 ± 1.63), recruited from the five public schools in Madeira’s capital (Funchal). Participants with pathologies and/or special educational needs were excluded from the study.

The participants and their legal representatives were introduced to the study, and informed consent was obtained. The study received ethical approval from the Scientific Committee of the Department of Physical Education and Sport at the University of Madeira (ACTA N.77, of 12 April 2016), respecting the declaration of Helsinki.

All the assessments were carried out by graduates in Physical Education and Sport who completed a training program. Following the training, a pilot study was conducted (*n* = 15 adolescents) to evaluate the test-retest reliability (one week apart), with the interclass correlation coefficient ranging from 0.797 (abdominal skinfold) to 0.999 (weight). 

The assessments took place at school, in a single session lasting approximately 30 min (questionnaire and CP). Cognitive function was assessed at two points: (i) at the start of the school year (September 2022) and (ii) at the end of the school year (May 2023). Between these two evaluations, physical activity levels were monitored using an accelerometer during a typical week.

### 2.2. Instruments

#### 2.2.1. Cognitive Performance

CP was assessed through structured interviews using the Cognitive Telephone Screening Instrument (COGTEL) [23,31,32,33], validated for adolescents [33]. THE COGTEL [23,31,32,33] includes the following cognitive tasks: (i) prospective memory, (ii) verbal short-term memory, (iii) verbal long-term memory, (iv) working memory, (v) verbal fluency, and (vi) inductive reasoning. The total score results from aggregating the six tasks (Table 1).

#### 2.2.2. Physical Activity

An ActiGraph accelerometer (GT3X+) was used to quantify PA, with analysis recorded at 60 s [34]. Time at different levels of PA was derived using ActiLife software (version 6).

The accelerometer was affixed to an elastic belt above the right iliac crest [34,35] throughout the day (from waking up to bedtime) for seven consecutive days (removed only for activities in contact with water, such as bathing). A typical week was evaluated (five working days and two weekend days) without holidays or school interruptions. 

All participants filled out a diary that contained: (i) the time they put on and took off the accelerometer, (ii) instances of non-usage, and (iii) organized physical activities, physical education, and/or sports (modality and duration). The diary was checked daily. Only participants who reported using the accelerometer for 10 or more hours per day in three or more days were included in the study. The variable used results from the average of the days evaluated.

#### 2.2.3. Socioeconomic Status

School social support was used as an indicator of socioeconomic status. The calculation of school social support is described in Rodrigues et al. [23], with participants classified into four categories. Income and the number of family members are used to calculate the school’s social support [23] with higher categories corresponding to higher family income. 

### 2.3. Data Analysis

The change in cognitive performance (∆CP) was calculated as the difference between the second and first moments of the CP dimension. The continuous variables were expressed as the mean, standard deviation, and frequency distribution for categorical data. Statistical normality was tested using the Kolmogorov–Smirnov test. To ascertain the variance between the second and first moments of the CP assessment, the Student’s T-test for the paired sample was employed. Bonferroni-corrected significance level was utilized to interpret this test (0.05/7 = 0.0071). The effect size for the change in CP from initial to final was determined using the Glass Delta, interpreted as trivial <0.35; small = 0.35–0.80; moderate = 0.80–1.50, and large >1.5 [36].

Analysis of covariance was used to examine the differences between sexes and levels of schooling in ∆CP, controlling for school social support. Multivariate analysis of variance was used to discern the differences between sex, level of schooling, and school social support across different levels of PA. Multiple linear regression was employed to analyze the influence of PA levels on ∆CP.

Statistical analyses were performed using SPSS version 29.0 statistical software for Windows (SPSS Inc., Chicago, IL, USA). The significance level adopted was 5%.

## 3. Results

The characterization of the sample is shown in Table 2. The majority of participants are female (57.9%), high school students (66.5%), and have low school social support (67.7%). Regarding the PA profile, sedentary behaviors predominate.

Between the two evaluation periods, there was an improvement in performance in all dimensions except for verbal fluency (−0.51 ± 12.61). The differences between the final and initial assessments were not significant in the prospective memory, working memory, verbal fluency, and verbal long-term memory dimensions (*p* > 0.007). The inductive reasoning and total score dimensions presented higher significance variation between the initial and final variables assessments, with an effect size of small magnitude (Table 3).

Through covariance analysis, there were no differences in ∆CP between sexes or between different social school supports (*p* > 0.05). However, there are differences between participants in middle and high school (F_(2_._359)_ = 7.56, *p* = 0.028). Participants in the middle school have a higher ∆CP value (5.51 ± 7.5 vs. 1.72 ± 6.75).

Regarding PA, in general, the sample presents sedentary behavior. On average, boys exhibit higher levels of vigorous (1.10% ± 0.67% vs. 0.70% ± 0.44%) and very vigorous (0.20% ± 0.28% vs. 0.06% ± 0.08%) PA (*p* < 0.05). Additionally, the year of schooling appears to impact PA (*p* < 0.05). High school participants have, on average, higher levels of sedentary activities (75.41 ± 2.76 vs. 74.00 ± 3.09), and those in the middle have moderate levels of activity (7.31 ± 2.08 vs. 6.12 ±2.01). Notably, there was no effect of school social support on the PA profile (*p* > 0.05).

Multiple linear regression was conducted to determine the influence of different levels of PA, age, sex, and school social support on the total score of CP. The independent variables collectively account for 17.2% of the variability of total score of CP (F_(3.362)_ = 14.261, *p* < 0.0001, R^2^ = 17.2%), age 9.3% (ß = −0.332, t = −4.255, *p* < 0.001, R^2^ = 9.3%), and vigorous and very vigorous PA explains 7.9% (ß = 0.283, t = 3.627, *p* < 0.001, R^2^ = 7.9%).

## 4. Discussion

This study examined the variation of CP in Portuguese adolescents in one school year and its relationship with PA. There was a positive evolution between the two assessments in the total score, with a small effect size. The fact that the total CP score is the product of several dimensions, some with significant effects and others without, could be a factor in reducing the magnitude of the effect of the total score. It is in the dimensions of verbal short memory and inductive reasoning that the magnitude of the effect is greatest, which may be supported by the developmental stage of our participants. Adolescence is a particular period of life in which cognitive development plays a crucial role. The brain undergoes greater specialization integration of structures, and, in general, there is a “refinement” of cognitive skills. Accordingly, the literature reports that despite inter-individual variability, children’s cognitive skills develop and improve with age, contributing to improved CP [37].

Furthermore, findings from this study indicated that the level of schooling influenced variation in CP, whereas neither gender nor school social support demonstrated a significant effect. These results appear to contradict the prevailing notion that socioeconomic status is associated with CP. Over the past few decades, extensive research has illuminated the impact of low socioeconomic status on cognitive functioning. It has been demonstrated that disparities in general intelligence (e.g., IQ scores) between children from high and low-income families emerge as early as late infancy and nearly treble by adolescence [38]. Additionally, socioeconomic status not only shapes cognitive function in these age groups but also predicts a decline in cognitive outcomes over time [39]. Moreover, contrary to expectations based on socioeconomic status, this study found no change in CP between the sexes. However, research on adolescents from low socioeconomic status has suggested that gender serves as a predictor of cognitive pathways, with CP improving in boys over time while decreasing in girls [39]. Conversely, a study conducted on adolescents in Spain revealed gender differences, with girls exhibiting stronger CP [40]. 

The existing literature indicates that older adolescents typically exhibit greater CP [40]. However, our study revealed a nuanced progression within high and middle school throughout the academic year, with participants from middle school demonstrating a higher ∆CP compared to participants from high school. This could be because while not as dramatic as the changes seen in childhood, cognitive abilities continue to develop and refine throughout the adolescent years, though at a slower rate compared to the rapid progress made during childhood [41]. Other studies have also suggested that some cognitive functions, such as cognitive flexibility, remain relatively stable after the preteen growth spurt. After this period, cognitive changes occur mainly in the functional integration [42]. This could help to explain why adolescents from middle school show higher ∆CP in our study.

Nevertheless, our search did not uncover any previous research illustrating such temporal, particularly over the duration of a school year. This highlights the need for more longitudinal studies aimed at comprehensively understanding the development of brain functions and structures over time, alongside the other potentially influencing factors. Additionally, literature has highlighted that since adolescents are enduring complex cognitive changes, exercise can lead to different responses in a teen brain compared to childhood or adulthood [43]. A “fine tuning” of the cognitive ability spans throughout adolescence because of processes such as pruning, myelination, and integration of cortical areas Thus, adequate cognitive development in this stage of life is vital for learning and can be affected by external factors. 

The literature has reported that physical fitness can indeed facilitate brain maturation and CP [44]. Studies have also shown links between PA and different brain structures, which can lead to hypothesizing that PA may influence cognitive functions [44]. PA has been consistently linked with enhanced CP [17,45]; hence, exercise during this important developmental stage can have an impact on neurological and cognitive outcomes [45]. However, determining the optimal level of PA to impact this relationship remains the subject of ongoing research [19,27]. 

Studies conducted on adolescents in Portugal have demonstrated the positive effects of active transportation on cognitive performance for periods exceeding thirty minutes [23], suggesting that light to moderate intensities may positively influence cognitive function. However, our findings suggest that only vigorous and very vigorous levels of physical activity seem to impact ∆CP. This aligns with previous studies on the types of PA, such as sports [22,25] and aerobic and anaerobic exercise [40], which predominantly involve higher intensities that can improve CP. Despite the results obtained, Jeppesen, et al. [46] advocate for light-intensity PA. Future interventions aimed at enhancing CP and, subsequently, academic performance ought to consider strategies that encourage vigorous and very vigorous PA.

The significant proportion of sedentary behavior among the participants cannot be overlooked despite the demonstrated benefits of high-intensity PA on CP. Adolescents are particularly susceptible to sedentary behavior, with smartphone usage being the major contributing factor, alongside prevailing lifestyle trends. By 2024, it is projected that 60.42% of the population will own smartphones, with adolescents constituting the highest percentage of users [47]. While some studies have identified a connection between smartphone use and certain cognitive domains (e.g., attentional capacities) [48], research into these effects remains limited, with scarce longitudinal evidence [49]. Although this behavior does not directly account for the variation in CP in this study, it is imperative to consider it in future studies.

As outlined above, the observed increase in CP from the beginning to the end of the school year can be attributed to the participant’s level of PA. However, it is essential to note that the COGTEL [31] evaluates multiple domains of cognitive performance, and only working memory, verbal short memory, prospective memory, and inductive reasoning showed improvement over time beyond the overall score. Syväoja, Kankaanpää [50] also affirmed the impact of PA on the working memory of adolescents. Working memory plays a crucial role in academic performance, particularly in mathematics, mediated by physical exercise. Exercise-induced increases in neurotransmitter release, such as dopamine, are pivotal for memory and attention processes [51]. Vigorous exercise increases blood flow to the brain, providing more oxygen and nutrients needed for the brain to function properly. This increased circulation stimulates the formation of new blood vessels and neuronal connections, which can improve cognitive function [27]. However, this mechanism for improving cognitive function may also be the result of the link between vigorous physical activity and a healthier lifestyle, such as sleep and a balanced diet, which contributes to CP. Our work has certain limitations despite the significant contributions it can offer to understanding the level of physical exercise that influences changes in CP. One limitation of the study is that it allowed us to determine the level of PA that increases CP but it did not explore whether this intensity had the same effects on the various domains that make up CP. Additionally, it lacks the possible influence of confounding factors, such as nutritional status, maturation, eating habits, and sleep patterns, on the relationship between PA and CP. Health status between the two assessments was also not controlled. This could potentially affect the CP. Furthermore, the use of convenience sampling to select participants limits the generalizability of the study findings to a broader population. These limitations underscore the need for further research to elucidate the relationship between physical activity and cognitive function across various domains.

## 5. Conclusions

This study showed that age and PA contribute to explaining variations in CP in adolescence. Many developmental changes occur with age, including cognitive abilities. However, external factors can also impact cognitive development.

PA has been shown in the literature to influence cognitive functions such as attention or memory and other executive functions. This study highlights this relationship particularly that intensive PA plays an important role in cognitive performance.

In this context, and in the face of an increasingly sedentary pediatric population, there is a clear need to design and develop programs to promote PA, especially vigorous and very vigorous. At the same time, it is necessary for intervention programs to consider different PA profiles according to adolescents’ development stages and their possible influence on cognitive development.

As physical education is the only organized PA that covers all students in school settings, it can be an excellent means of intervention and promotion of physical activity at higher intensities, which should be considered in the development of future intervention programs and to promote CP.

## Figures and Tables

**Table 1:** Cognitive performance, task, and score.

Cognitive Performance	Task	Score
Prospective memory	Quantified with an event-based task. Intention is formed at the beginning of the interview;	0 or 1
Verbal short memory	Word pair association test, with immediate recall;	0 to 8
Verbal long-term memory	With the same pairs of words as in the short-term verbal memory test but with a delayed retrieval at the end of the interview;	0 to 8
Working memory	Report in reverse order using a sequence of numbers;	0 to 12
Verbal fluency	Indicate the largest number of words beginning with a given letter in 1 min;	0 to unlimited
Inductive reasoning	Based on a sequence of 5 numbers, built according to a mathematical rule, the participants had to indicate the sixth number in the sequence;	0 to 8
Total score	Total score = 7.2 × prospective memory + 1.0 × verbal short-term memory + 0.9 × verbal long-term memory + 0.8 × working memory + 0.2 × verbal fluency + 1.7 × inductive reasoning score	-

**Table 2:** The adolescents’ characteristics (*n* = 366).

		Total
Sex	Men, *n* (%)	154 (42.1%)
	Women, *n* (%)	212 (57.9%)
Age	Age (years)	15.5 ± 1.6
Scholarly	Middle school, *n* (%)	123 (33.6%)
	High school, *n* (%)	243 (66.5%
School social support	1 (low). *n* (%)	174 (47.5%)
	2. *n* (%)	74 (20.2%)
	3. *n* (%)	36 (9.8%)
	4 (high). *n* (%)	82 (22.4%)
Physical Activity (%)	Sedentary (%)	74.8 ± 2.9
	Light (%)	17.7 ± 1.5
	Moderate (%)	6.6 ± 2.1
	Vigorous (%)	0.8 ± 0.5
	Very Vigorous (%)	0.1 ± 0.2
Cognitive Performance	Prospective memory	0.8 ± 0.40
	Verbal short memory	4.86 ± 1.83
	Working memory	6.20 ± 2.03
	Verbal fluency	30.84 ± 9.69
	Inductive reasoning	4.08 ± 1.77
	Verbal long-term memory	5.82 ± 1.75
	Total Score	36.70 ± 9.75

Note: Values are means ±  standard deviations number and proportions (%) for categorical data.

**Table 3:** Cognitive performance (*n* = 366).

Cognitive Performance	IA	FA	∆CP	T	*p*	Glass’s Delta
Prospective memory (*n*)	0.86 ± 0.35	0.91 ± 0.29	0.05 ± 0.29	2.628	0.009	0.14
Verbal short memory (*n*)	4.86 ± 0.83	5.32 ± 1.59	0.41 ± 1.99	3.395	0.001	0.55
Working memory (*n*)	6.20 ± 2.02	6.64 ± 2.25	0.32 ± 2.04	2.633	0.009	0.22
Verbal fluency (*n*)	30.84 ± 9.68	29.45 ± 11.26	−0.51 ± 12.61	−0.689	0.497	−0.14
Inductive reasoning (*n*)	4.08 ± 1.76	4.81 ± 1.88	0.62 ± 1.62	6.330	<0.0001	0.41
Verbal long-term memory (*n*)	5.82 ± 1.75	6.1 ± 1.56	0.18 ± 1.74	1.663	0.097	0.16
Total score (*n*)	36.69 ± 9.75	40.28 ± 9.95	2.64 ± 7.14	6.135	<0.0001	0.37

IA—Initial Assessment; FA—Final Assessment; ∆CP = FA − IA.

## Data Availability

The data presented in this study are available on request from the corresponding author.
